# Association of Umbilical Cord Coiling Index With Pregnancy Outcomes in the Second Trimester: A Prospective Observational Study

**DOI:** 10.1002/hsr2.73251

**Published:** 2026-09-25

**Authors:** Somayeh Barake, Poria HoseiniAliabadi, Seyed Mohammad Hassan Hosseiny, Mehradad Nabahati, Rahele Mehraeen

**Affiliations:** ^1^ Radiology Ward, Sina Hospital Semnan Iran; ^2^ Department of Radiology Babol University of Medical Sciences Babol Iran

**Keywords:** Apgar score, low birth weight, oligohydramnios, perinatal outcomes, umbilical cord index

## Abstract

**Background and Aims:**

The umbilical cord coiling index (UCI) is proposed as a predictor of adverse pregnancy outcomes. However, studies show varying results regarding UCI and prenatal outcomes, particularly between 18 and 24 weeks, indicating a need for further investigation.

**Methods:**

This prospective observational study was conducted at Ayatollah Rouhani Teaching Hospital, Babol, Iran, between 2021 and 2024. Antenatal Umbilical Cord Coiling Index (aUCI) was measured sonographically at 18–24 weeks of gestation, and participants were followed until delivery to record maternal and neonatal outcomes. Multivariable logistic regression was applied to adjust for potential confounders. SPSS version 22 was used for data analysis.

**Results:**

Of 422 women initially enrolled, 410 completed follow‑up and were analyzed. The mean antenatal UCI was 0.58 ± 0.14 coils/cm. Based on percentile thresholds, 14.9% were hypocoiled, 73.0% normocoiled, and 12.2% hypercoiled. Perinatal outcomes differed significantly across groups, with higher rates of low birth weight, oligohydramnios, low Apgar score, and preterm labor in abnormal coiling groups compared with normocoiled cords (*p* < 0.05). Regression analysis showed that hypercoiling was independently associated with premature rupture of membranes (OR = 2.65), low Apgar score (OR = 3.31), oligohydramnios (OR = 5.62), and low birth weight (OR = 3.06). Hypocoiling did not demonstrate significant associations with these outcomes (*p* > 0.05).

**Conclusion:**

Hypercoiled cords were associated with an increased risk of LBW, oligohydramnios, low Apgar scores, and PROM. Early UCI evaluation could improve prenatal care by identifying at‐risk pregnancies.

## 1 Introduction

Optimal umbilical cord function is essential for appropriate fetal development and overall well‐being [1]. Umbilical cord abnormalities including atypical insertion patterns, structural twists, true knots, and even rare complications such as cord hematoma can collectively disrupt fetoplacental circulation and pose risks for adverse perinatal outcomes [2]. The hallmark of the umbilical cord is its helical coiling, which arranges the vessels in a protective spiral configuration [3]. Umbilical vessel coiling begins to develop by approximately 28 days after conception and is established in nearly 95% of fetuses by around 9 weeks and the helical pattern of the cord can be detected on ultrasonography as early as the first trimester [4]. The degree of umbilical cord coiling is quantified by the umbilical cord coiling index (UCI), defined as the number of complete coils per centimeter of cord length [5]. On prenatal ultrasound, UCI can be assessed to classify cords as hypercoiled or hypocoiled, Such abnormal umbilical cord coiling disrupts fetal blood flow and is associated with adverse neonatal outcomes [6]. Evaluation of the umbilical cord coiling index is most reliable in the late second trimester, when the relatively greater volume of amniotic fluid in proportion to fetal size allows clearer visualization of longer cord segments [7]. Abnormal UCI, either hypocoiled or hypercoiled, has been associated with fetal growth restriction, preterm birth, low Apgar scores, and increased medical interventions [8]. However, studies examining UCI during 18–24 weeks of gestation have shown inconsistent results. Understanding the predictive value of UCI during this period may help identify at‐risk pregnancies and improve prenatal care. This study aims to evaluate the relationship between UCI measured by ultrasound at 18–24 weeks and adverse prenatal outcomes.

## 2 Materials and Methods

### 2.1 Study Design and Patients

This study was designed as a prospective observational study and conducted at Ayatollah Rouhani Teaching Hospital, Babol, Iran, between 2021 and 2024. All eligible participants who met the predefined inclusion and exclusion criteria during the study period were enrolled using consecutive sampling. Consequently, a total of 422 pregnant women were initially enrolled.

Pregnant women between 18 and 24 weeks of gestation who attended the hospital for routine prenatal care and intended to deliver at the same center were eligible for enrollment.

*Inclusion criteria included:* singleton pregnancy, gestational age 18–24 weeks, presence of a three‐vessel umbilical cord, and provision of informed consent.

*Exclusion criteria included:* multiple pregnancy, fetal anomalies, maternal comorbidities such as diabetes or hypertension, umbilical cord or placental anomalies, inability to follow‐up until delivery, maternal history of smoking or substance abuse, recurrent miscarriage (> 3 times), or history of complicated deliveries.

**Figure 1:**
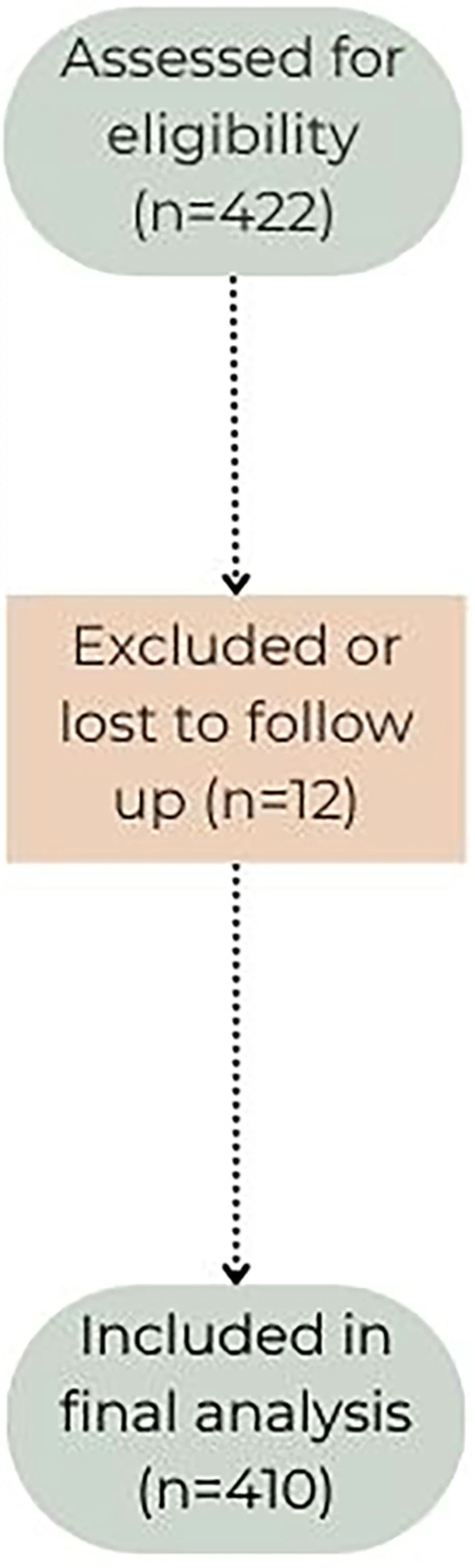
Study flowchart illustrating the enrollment, exclusion, and final inclusion of the study participants.

Of the 422 women initially enrolled, 12 were excluded during follow‐up due to incomplete records or loss to follow‐up, leaving 410 participants for the final analysis. A detailed study flowchart illustrating the inclusion, exclusion, and follow‐up process is presented in Figure 1.

### 2.2 Ultrasound Technique and Measurements

**Figure 2:**
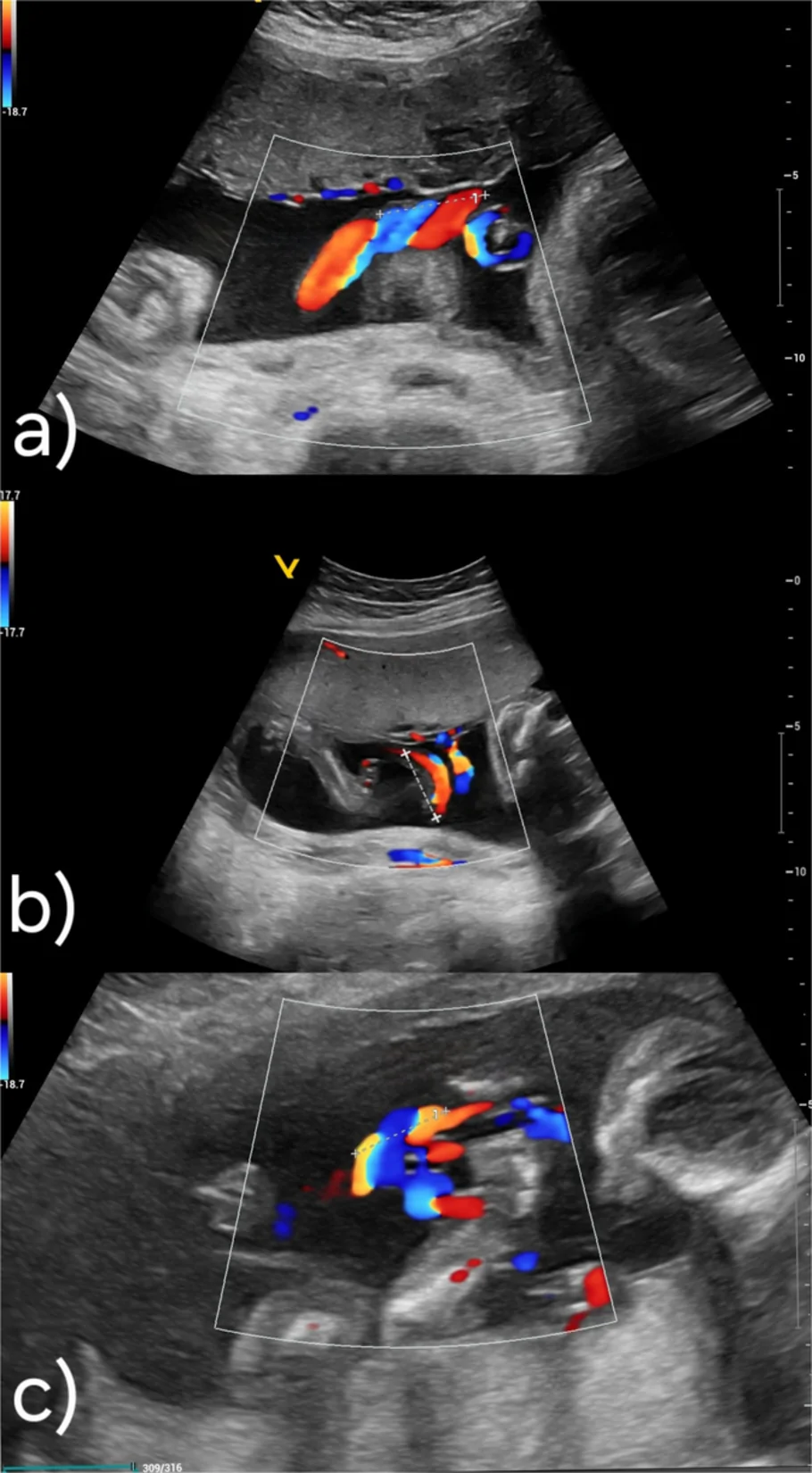
Representative color Doppler ultrasound images demonstrating the technique for antenatal assessment of the umbilical cordcoiling index: (a) normocoiled, (b) hypocoiled, and (c) hypercoiled umbilical cords.

At baseline (18–24 weeks of gestation), the Umbilical cord Coiling Index (aUCI) was measured using a Mindray DC‐8 Expert ultrasound system by a single experienced radiology faculty member to ensure consistency. A free‐floating segment of the umbilical cord in the amniotic fluid was carefully selected. Color Doppler imaging was routinely applied to enhance the visualization of the umbilical vessels and accurately distinguish the vascular coils. The number of complete vascular coils (one complete 360° spiral of the umbilical arteries around the umbilical vein) per centimeter of cord length was calculated (Figure 2 shows the sonographic visualization of the cord using Color Doppler). While Color Doppler was utilized for structural visualization, Doppler velocimetry indices (e.g., Resistive Index (RI), Pulsatility Index (PI), S/D ratio) were not recorded as they were outside the scope of this index calculation. All participants were then prospectively followed until delivery.

### 2.3 Definitions and Important Variables

#### 2.3.1 Baseline Clinical and Demographic Variables

Key maternal variables including maternal age (years), body mass index (BMI, calculated as kg/m^2^), gravida (number of pregnancies, recorded as 0, 1, 2, or 3), mode of delivery (cesarean/vaginal delivery), and maternal history of abortion (yes/no) were recorded for all participants.

#### 2.3.2 Classification of Umbilical Cord Coiling

According to the antenatal Umbilical cord Coiling Index (aUCI) measured within our specific study population, participants were divided into three categories: hypocoiled (aUCI below the 10th percentile), normocoiled (aUCI between the 10th and 90th percentiles), and hypercoiled (aUCI above the 90th percentile) [9].

#### 2.3.3 Adverse Perinatal Outcomes (Dependent Variables)

Included low birth weight (< 2500 g), premature rupture of membranes (PROM, rupture of the fetal membranes prior to the onset of labor), low Apgar score (< 7 at 5 min), and oligohydramnios (amniotic fluid index ≤ 5 cm or the single deepest pocket < 2 cm).

### 2.4 Ethical Consideration

The protocol of this study was approved by the Ethics Committee of Babol University of Medical Sciences (IR.MUBABOL.REC.1399.048). Written informed consent was obtained from all participants prior to enrollment. Ethical principles following relevant guidelines and regulations were considered by researchers at all stages of the study.

### 2.5 Statistical Analysis

Data were analyzed using SPSS version 22 (IBM Corp., Armonk, NY, USA). Descriptive statistics were reported as frequency, percentage, mean, and standard deviation. Comparisons between groups were performed using the Chi‐square test for categorical variables and continuous variables were compared using the Kruskal–Wallis test. Multivariable logistic regression was performed to assess the association between umbilical cord coiling categories and adverse perinatal outcomes. Odds ratios (ORs) and 95% confidence intervals (CIs) were reported. A *p* < 0.05 was considered statistically significant.

## 3 Results

Of the 422 women initially enrolled, 12 were excluded during follow‑up due to incomplete records or loss to follow‑up, leaving 410 participants for final analysis. The mean maternal age was 29.03 ± 6.03 years, and the mean maternal BMI was 25.27 ± 2.87 kg/m^2^. The mean aUCI was 0.58 ± 0.14 coils/cm, with values ranging from 0.30 to 1.10 coils/cm. In the study population, the 10th and 90th percentiles of aUCI corresponded to 0.41 and 0.76 coils/cm, respectively. Based on these thresholds, 61 women (14.9%) were classified as hypocoiled, 299 (73.0%) as normocoiled, and 50 (12.2%) as hypercoiled (Table 1).

**Table 1:** Maternal and baseline characteristics across UCI groups.

Variables	Total *N* (%) = 410 (100)	Hypocoiled *N* (%) = 61 (14.9)	Normocoiled *N* (%) = 299 (73)	Hypercoiled *N* (%) = 50 (12.2)	Sig.^a^
Maternal age, years (mean ± SD)	29.03 ± 6.03	28.67 ± 0.7	29.1 ± 0.4	29.06 ± 0.83	0.15
BMI, kg/m^2^ (mean ± SD)	25.27 ± 2.87	24.91 ± 2.53	25.34 ± 2.91	24.44 ± 3.04	0.57*
Mode of delivery, *n* (%)	Cesarean	32 (52.5)	145 (48.5)	23 (46)	0.78*
	Vaginal	29 (47.5)	154 (51.5)	27 (54)	
Gravida, *n* (%)	0	24 (39.3)	114 (38.1)	32 (64)	**0.003** **
	1	28 (45.9)	140 (46.8)	16 (32)	
	2	8 (11.5)	41 (13.7)	2 (4)	
	3	2 (3.3)	4 (1.3)	(0)	
History of abortion, *n* (%)	No	53 (86.9)	226 (75.6)	44 (88)	0.94**
	Yes	8 (13.3)	73 (24.4)	6 (12)	
LBW, *n* (%)	No	60 (98.4)	279 (93.3)	41 (82)	**0.003** **
	Yes	1 (1.6)	20 (6.7)	9 (18)	
Oligohydramnios, *n* (%)	No	58 (95.1)	274 (91.6)	33 (66)	**< 0.001** **
	Yes	3 (4.9)	25 (8.4)	17 (34)	
Low Apgar score, *n* (%)	No	60 (98.4)	278 (93)	40 (80)	**0.001** **
	Yes	1 (1.6)	21 (7)	10 (20)	
Preterm labor, *n* (%)	No	59 (96.7)	267 (88.3)	37 (74)	**0.001** **
	Yes	2 (3.3)	35 (11.7)	13 (26)	

Abbreviations: BMI, body mass index; LBW, low birth weight.

a Significant values (< 0.05) are shown in bold.

* *p* values were calculated using *χ* ^2^ test or Fisher's exact test as appropriate based on expected cell counts

** *p* values were calculated using the Kruskal–Wallis test for non‐normally distributed continuous variables.

Comparison of perinatal outcomes across the three UCI groups showed significant differences in several variables. The incidence of low birth weight, oligohydramnios, low Apgar score, and preterm labor was significantly higher in the hypocoiled and hypercoiled groups compared with normocoiled cords (*p* < 0.05). In contrast, no significant differences were observed in maternal age (*p* = 0.93), maternal weight (*p* = 0.61), history of abortion (*p* = 0.94), or mode of delivery (*p* = 0.78) among the groups. Detailed results are presented in Table 1.

Multiple regression analysis was performed to evaluate the association between maternal demographic factors and the risk of umbilical cord coiling abnormalities. The results indicated that most maternal demographic factors were not significantly associated with the umbilical cord coiling index. However, nulliparity was significantly related to an increased risk of hypercoiled umbilical cord compared to women with more than two pregnancies (OR = 5.53, *p* = 0.02) (Table 2).

**Table 2:** Association between maternal characteristics and abnormal umbilical cord coiling (multivariable regression model).

	Variables		OR	95% CI	Sig.^a^
Hypocoiled risk	History of abortion	No	2.17	0.98–4.82	0.06*
		Yes		reference	
	Gravida	0	0.9	0.38–21.3	0.81*
		1	0.9	0.4–2.08	0.81*
		> 2**		reference	
Hypercoiled risk	History of abortion	No	1.9	0.77–4.72	0.16*
		Yes		reference	
	Gravida	0	5.53	1.26–24.29	**0.02** *
		1	2.36	0.52–10.72	0.27*
		> 2**		reference	

a Significant values (< 0.05) are shown in bold.

* *p* values were derived from the multivariable logistic regression model using the Wald test.

** Due to the small number of people with three pregnancies (6 people), these people are shown in the form of 2 or more in the table.

Multivariable logistic regression analysis was performed to evaluate the association between umbilical cord coiling abnormalities and prenatal outcomes. Hypercoiled umbilical cord was significantly associated with an increased risk of PROM (OR = 2.65, 95% CI: 1.28–5.46, *p* = 0.008), low Apgar score (OR = 3.31, 95% CI: 1.45–7.53, *p* = 0.004), oligohydramnios (OR = 5.62, 95% CI: 2.75–11.49, *p* < 0.001), and low birth weight (OR = 3.06, 95% CI: 1.30–7.18, *p* = 0.01). In contrast, hypocoiled cords were not significantly associated with these outcomes (Table 3).

**Table 3:** Multivariable logistic regression analysis of UCI and adverse pregnancy outcomes.

	Outcome	OR	95% CI	Sig.^a^
Hypercoiled risk	PROM	2.65	1.28–5.46	**0.008** *
	Low Apgar score	3.31	1.45–7.53	**0.004** *
	Oligohydramnios	5.62	2.75–11.49	**< 0.001** *
	LBW	3.06	1.30–7.18	**0.01** *
Hypocoiled risk	PROM	0.256	0.06–1.09	0.07
	Low Apgar score	0.221	0.02–1.67	0.14
	Oligohydramnios	0.565	0.16–1.93	0.36
	LBW	0.233	0.03–1.76	0.16

Abbreviations: LBW, low birth weight; PROM, premature rupture of membranes.

a Significant values (< 0.05) are shown in bold.

* *p* values were derived from the multivariable logistic regression model using the Wald test.

## 4 Discussion

This study was designed to investigate the association between the umbilical cord coiling index (UCI) measured at 18–24 weeks of gestation and subsequent prenatal outcomes. Our findings demonstrated that hypercoiled cords were significantly associated with increased risks of low birth weight, PROM, low Apgar scores, and oligohydramnios, whereas hypocoiled cords did not show significant associations with these outcomes.

When compared with previous studies, our results are largely consistent with evidence suggesting that abnormal coiling, particularly hypercoiling, predisposes to adverse perinatal outcomes. Mishra and colleagues in India, with a larger sample size (*n* = 1200), similarly reported significant associations between hypercoiling and low birth weight as well as low Apgar scores, supporting the robustness of our findings [9]. Sharma and colleagues (2012) and Chitra et al. (2012) also observed increased frequencies of low birth weight in both hypercoiled and hypocoiled groups, although their analyses were limited by smaller sample sizes and less advanced imaging approaches [10, 11]. Studies such as Ohno and colleagues (2016, Japan) and Milani et al. (Iran, 2018), which evaluated coiling postpartum or in late pregnancy, did not find significant associations with low birth weight [12, 13], while Adesina and colleagues (2017, Nigeria), assessing cords after delivery, similarly reported no relationship with Apgar scores or oligohydramnios in either hypercoiled or hypocoiled groups [14]. Similarly, Takita et al. (2020, Japan), in a large postpartum cohort, reported no associations between hypocoiled cords and outcomes such as low birth weight, Apgar scores, and oligohydramnios [15], findings that parallel three of the outcomes examined in our study. Such inconsistencies across studies may be explained by differences in study design (antenatal vs. postpartum assessment), variation in diagnostic criteria for coiling abnormalities, and heterogeneity in study populations.

The heterogeneity across studies is notable. For example, Aanandini et al. reported no significant associations between hypercoiling and oligohydramnios or PROM [16], while our study found strong correlations, particularly with oligohydramnios (OR = 5.62). These discrepancies may be explained by differences in gestational age at examination, diagnostic criteria, and imaging modalities. Our use of color Doppler in the second trimester likely enhanced diagnostic accuracy compared to grayscale ultrasound or postpartum cord evaluation, which may underestimate antenatal coiling abnormalities. Although umbilical artery Doppler indices were not evaluated in the present study, previous investigations have demonstrated significantly higher umbilical artery resistance index (RI‐UA) in pregnancies with abnormal umbilical cord coiling. Increased RI‐UA has also been associated with lower birth weight and improved prediction of small for gestational age neonates [17]. These findings provide a plausible hemodynamic explanation for the association between hypercoiling and adverse pregnancy outcomes observed in our study and support the hypothesis that increased placental vascular resistance may contribute to fetal compromise. From a clinical perspective, early identification of abnormal coiling patterns may help anticipate conditions such as oligohydramnios or fetal growth restriction, in which induction of labor could be considered [18].

Meta‐analytic evidence, such as that summarized by Pergialiotis and colleagues, supports the conclusion that abnormal UCI, whether hypo‐ or hypercoiled, is associated with at least one adverse pregnancy outcome [19]. However, our findings suggest that hypercoiling may represent a more clinically significant abnormality, given its consistent association with multiple adverse outcomes. This aligns with Sharma and colleagues, who reported increased risk of low Apgar scores in hypercoiled cords [20], and Mittal et al. (2015), who found associations between both hypo‐ and hypercoiling with low birth weight [21]. In summary, our findings highlight hypercoiling of the umbilical cord as a significant predictor of adverse pregnancy outcomes, likely mediated through increased vascular resistance and impaired fetal blood flow. By contrast, hypocoiling did not show consistent associations, which may reflect a milder hemodynamic impact or variability in diagnostic definitions.

The main limitations of this study are its single‐center design and relatively small sample size, which may restrict the generalizability of the findings. The small sample size may also have contributed to wider CIs and reduced the precision of the estimated associations. In addition, the absence of umbilical artery Doppler indices limited the assessment of the hemodynamic mechanisms underlying abnormal cord coiling. Furthermore, the observational nature of the study limits causal interpretation of the observed associations.

## 5 Conclusion

In this prospective observational study, antenatal assessment of the umbilical cord coiling index (UCI) revealed strong adjusted associations between hypercoiling and clinically relevant adverse pregnancy outcomes. However, hypocoiling did not demonstrate consistent associations, suggesting a potentially milder hemodynamic impact or variability in diagnostic thresholds. Despite methodological differences across populations and assessment techniques, our findings contribute to the growing body of literature supporting UCI as a prognostic marker. Future investigations should employ standardized diagnostic criteria, integrate advanced imaging modalities, and explore underlying pathophysiological mechanisms such as alterations in umbilical blood flow and vascular resistance, in order to better define the clinical utility of UCI in routine obstetric care.

## Author Contributions

**Somayeh Barake:** data curation, conceptualization, project administration. **Poria HoseiniAliabadi:** formal analysis, software, writing – original draft, writing – review and editing, conceptualization. **Seyed Mohammad Hassan Hosseiny:** conceptualization, data curation, writing – original draft. **Mehradad Nabahati:** investigation, validation, methodology. **Rahele Mehraeen:** software, formal analysis, supervision, resources, investigation, funding acquisition, validation, visualization. All authors have read and approved the final version of the manuscript.

## Funding

The authors have nothing to report.

## Ethics Statement

The protocol of this study was approved by the Ethics Committee of Babol University of Medical Sciences (IR.MUBABOL.REC.1399.048).

## Consent

Written informed consent was obtained from all participants prior to enrollment.

## Conflicts of Interest

The authors declare no conflicts of interest.

## 1 Transparency Statement

The corresponding author (Rahele Mehraeen) affirms that this manuscript is an honest, accurate, and transparent account of the study being reported; that no important aspects of the study have been omitted; and that any discrepancies from the study as planned (and, if relevant, registered) have been explained.

## Data Availability

The data that support the findings of this study are available from the corresponding author upon reasonable request. Rahele Mehraeen had full access to all of the data in this study and takes complete responsibility for the integrity of the data and the accuracy of the data analysis.
